# Risk factors associated with solid oral tumors in dogs: a case–control study in São Paulo, Brazil

**DOI:** 10.3389/fvets.2026.1738835

**Published:** 2026-03-02

**Authors:** Marcella Collaneri Carrilho, Katia Pinello, Helena Sofia Salgueiro, Laura Sichero, Denner Santos dos Anjos, Guilherme da Silva Rogerio, Andrigo Barbosa de Nardi, Maria Lucia Zaidan Dagli

**Affiliations:** 1 Laboratory of Experimental and Comparative Oncology, School of Veterinary Medicine and Animal Science, University of São Paulo, São Paulo, Brazil; 2 Epidemiology Unit (EPIUnit), Institute of Public Health of the University of Porto (ISPUP), Porto, Portugal; 3 Center for Translational Research in Oncology, Instituto do Câncer do Estado de São Paulo-ICESP, Hospital das Clínicas da Faculdade de Medicina da Universidade de São Paulo FMUSP HC, São Paulo, SP, Brazil; 4 Eletro-Onkovet Service, Franca, SP, Brazil; 5 Department of Veterinary Clinic and Surgery, Universidade Estadual Paulista (UNESP), Jaboticabal, Brazil

**Keywords:** dogs, fibrosarcoma, melanoma, neoplasms, oral neoplasms, risk factors

## Abstract

**Introduction:**

Solid oral tumors represent approximately 6% of malignant neoplasms in dogs, with melanoma, squamous cell carcinoma, and fibrosarcoma identified as the most prevalent types. This case-control study examined internal and external factors associated with their development.

**Methods:**

Owners of 80 dogs with oral tumors and 95 healthy controls completed a structured epidemiological questionnaire. Univariate and multivariate analyses were performed.

**Results:**

Significant associations were identified regarding age, weight, reproductive status, dietary habits, and environmental exposures, such as barbecue smoke and professional dental cleaning.

**Discussion:**

These findings suggest potential risk factors, though the study’s observational design requires cautious interpretation. Further investigation through prospective studies is warranted.

## 1 Introduction

The incidence of cancer in dogs has increased substantially, largely due to greater canine longevity (1). Cancer, a multifactorial disease, is now the primary cause of death in dogs and cats, underscoring the importance of veterinary oncology and cancer epidemiology (2). The most frequent malignant oral neoplasms in dogs are melanomas, squamous cell carcinomas, and fibrosarcomas (3–5).

Malignant oral melanomas, which originate from melanocytic cells, are the most common oral tumors in dogs, particularly in elderly dogs of pigmented breeds such as Chow Chows, Cocker Spaniels, and Poodles. Squamous cell carcinomas arise from keratinocytes, while fibrosarcomas, of mesenchymal origin, are also prevalent and display variable invasiveness (6, 25). Although internal factors such as genetic mutations and immune dysfunction contribute to tumorigenesis, external influences, including environmental exposures, are also critical.

Approximately 6% of malignant neoplasms in dogs occur in the oral cavity, making it the fifth most common site of tumor development (4, 7, 8). A survey of 1,813 canine tumors diagnosed between 1993 and 2002 at the Animal Pathology Service of the University of São Paulo showed that oral squamous cell carcinoma accounted for 5% of epithelial tumors (9). Oral cavity tumors and tumor-like lesions are often detected during routine examinations. These lesions may cause pain, discomfort, or anorexia, but can also be incidental findings, frequently associated with periodontal disease. Owners often present dogs for evaluation due to sialorrhea, halitosis, or mastication difficulties (10). In early stages, these neoplasms may be misdiagnosed as inflammatory processes like gingivitis or stomatitis, highlighting the importance of comprehensive clinical examination.

Definitive diagnosis of solid oral tumors requires histopathological confirmation via incisional or excisional biopsy. Persistent irritation and chronic inflammation from conditions like chronic gingivitis may promote malignant transformation. Additional proposed risk factors include passive exposure to tobacco smoke, use of flea-control collars, and dietary or environmental influences (11). Specifically, the rationale for examining exposures to indoor pollutants like incense and water source composition stems from their documented carcinogenic potential in humans and other species, where they are linked to mucosal irritation and oxidative stress (12, 13). This study aims to identify key epidemiological and etiological factors, including these specific environmental and lifestyle variables, associated with solid oral tumors in dogs.

## 2 Materials and methods

### 2.1 Study design and population

This observational case–control study investigated risk factors associated with solid oral tumors in dogs. The study population was recruited from 30 veterinary hospitals and clinics in São Paulo state, Brazil, selected to reflect diverse geographical areas and practice types. Cases were defined as dogs with histopathologically confirmed solid oral tumors. Controls were dogs examined at the same clinics during the study period who showed no evidence of oral tumors. To reduce confounding, individual matching was performed, where each control was matched to a case by age (within 1 year), breed (similar size and morphology), and sex.

Data were collected through a structured epidemiological questionnaire administered online via Google Forms^1^. The questionnaire was developed based on previously established environmental and lifestyle risk factors in veterinary oncology and was pre-tested with a pilot group of 10 owners to ensure clarity and reproducibility of the questions. Information collected included animal demographics (sex, breed, age), lifestyle habits (diet, water source, history of periodontal disease), environmental exposures (passive tobacco smoke, incense use, proximity to barbecue smoke, biomass burning, and magnetic fields), and owner characteristics (residence type, address, and smoking status).

### 2.2 Statistical analysis

Before inferential analyses, the dataset was reviewed for missing values and inconsistencies. Dogs with non-solid tumors or incomplete medical records were excluded to ensure data integrity. Only variables with sufficient observations for statistical analysis were included. All analyses were performed using Excel® (Microsoft Corporation), SPSS (IBM), and R version 4.1.2. Normality of continuous variables was tested using the Shapiro–Wilk test. Because age and weight were not normally distributed (*p* < 0.05), the Wilcoxon rank-sum test (Mann–Whitney U test) was used to compare medians between cases and controls.

Categorical variables were reported as counts and percentages, and continuous variables as medians with interquartile ranges (IQR). To identify risk factors, univariate logistic regression was performed for each independent variable, with *p* < 0.05 considered statistically significant. Variables significant in the univariate analysis were entered into a multivariate logistic regression model to adjust for confounders. Multicollinearity among independent variables was assessed using the Variance Inflation Factor (VIF) to ensure model stability. Stepwise selection guided by the Akaike Information Criterion (AIC) optimized the final model. Adjusted odds ratios (aOR) with 95% confidence intervals (CI) were reported, where aOR > 1 indicated increased risk and aOR < 1 indicated a protective effect. Model fit was evaluated using the likelihood ratio test.

### 2.3 Ethical considerations

This study adhered to ethical guidelines for animal research. Informed consent was obtained from all pet owners before data collection. Ethical approval was granted by the Ethics Committee on the Use of Animals (CEUA), School of Veterinary Medicine and Animal Science, University of São Paulo (Approval No.: 6827260123).

## 3 Results

### 3.1 Tumor types

**Figure 1:**
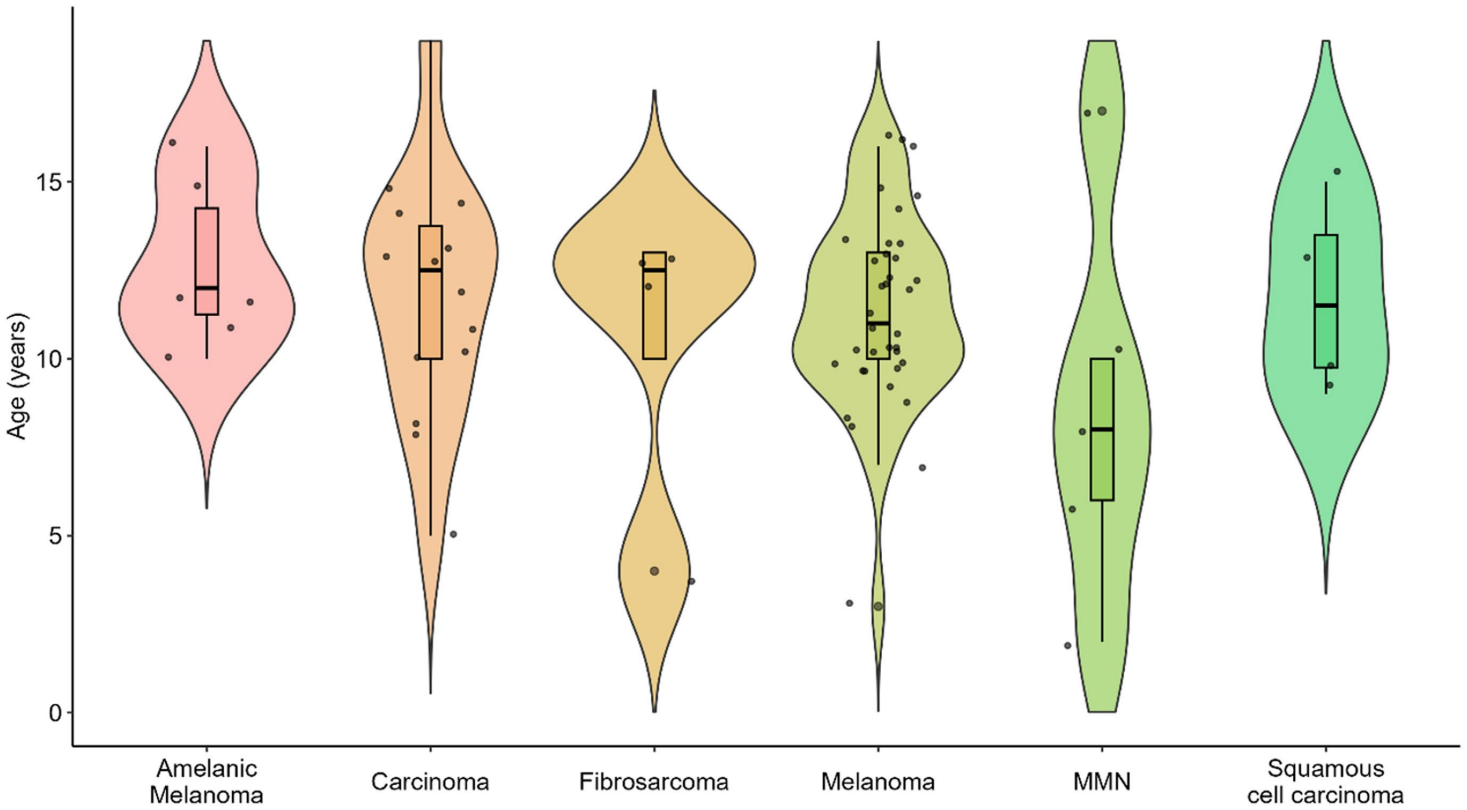
Types of solid oral tumors in dogs included in the study (*n* = 80).

Table 1 presents the distribution of oral tumor types in the dogs included in this study. Among the 80 analyzed cases, melanoma was the most common (*n* = 36, 39.1%), followed by squamous cell carcinoma (*n* = 14, 15.2%) and fibrosarcoma (*n* = 4, 4.3%). Less frequent diagnoses included amelanotic melanoma, carcinoma, and malignant mesenchymal neoplasia. No statistically significant differences in age distributions were observed across the various tumor types (*p* > 0.05, ANOVA). Figure 1 illustrates the relative frequency of these oral tumor types.

**Table 1:** Oral tumor types in the sampled dogs.

Tumor	Type	N	%	Average Age	Median Age	Min Age	Max Age	Standard Deviation (SD)
1	Acantomatous ameloblastoma	1	0.6	11.00	11.0	11	11	*NA*
2	Squamous cell carcinoma	4	2.3	11.75	11.5	9	15	2.75
3	Carcinoma, NOS	14	8.0	11.79	12.5	5	19	3.49
4	Condrosarcoma	1	0.6	8.00	8.0	8	8	*NA*
5	Ossifying epulides	1	0.6	3.00	3.0	3	3	*NA*
6	Fibrosarcoma	4	2.3	10.50	12.5	4	13	4.36
7	Hemangiosarcoma	2	1.1	14.50	14.5	13	16	2.12
8	Mast cell tumor	3	1.7	9.33	6.0	6	16	5.77
9	Melanoma	36	20.6	11.31	11.0	3	16	2.71
10	Amelanotic melanoma	6	3.4	12.67	12.0	10	16	2.34
11	NA	1	0.6	10.00	10.0	10	10	*NA*
12	Malignant mesenchymal neoplasia	5	2.9	8.60	8.0	2	17	5.55
13	Papilloma	1	0.6	1.00	1.0	1	1	*NA*
14	Plasma cell tumor	1	0.6	9.00	9.0	9	9	*NA*
15	Sarcoma	1	0.6	8.00	8.0	8	8	*NA*
16	*TOTAL*	94	53.7	7.99	7.5	1	18	4.15

SD, standard deviation; Min, minimum; Max, maximum; NA, not applicable.

### 3.2 Descriptive analysis and univariate logistic regression

A total of 80 cases (dogs with histologically confirmed solid oral tumors) and 95 controls (dogs without oral tumors) were included in the final analysis. Owners of all 175 dogs completed the epidemiological questionnaire.

**Figure 2:**
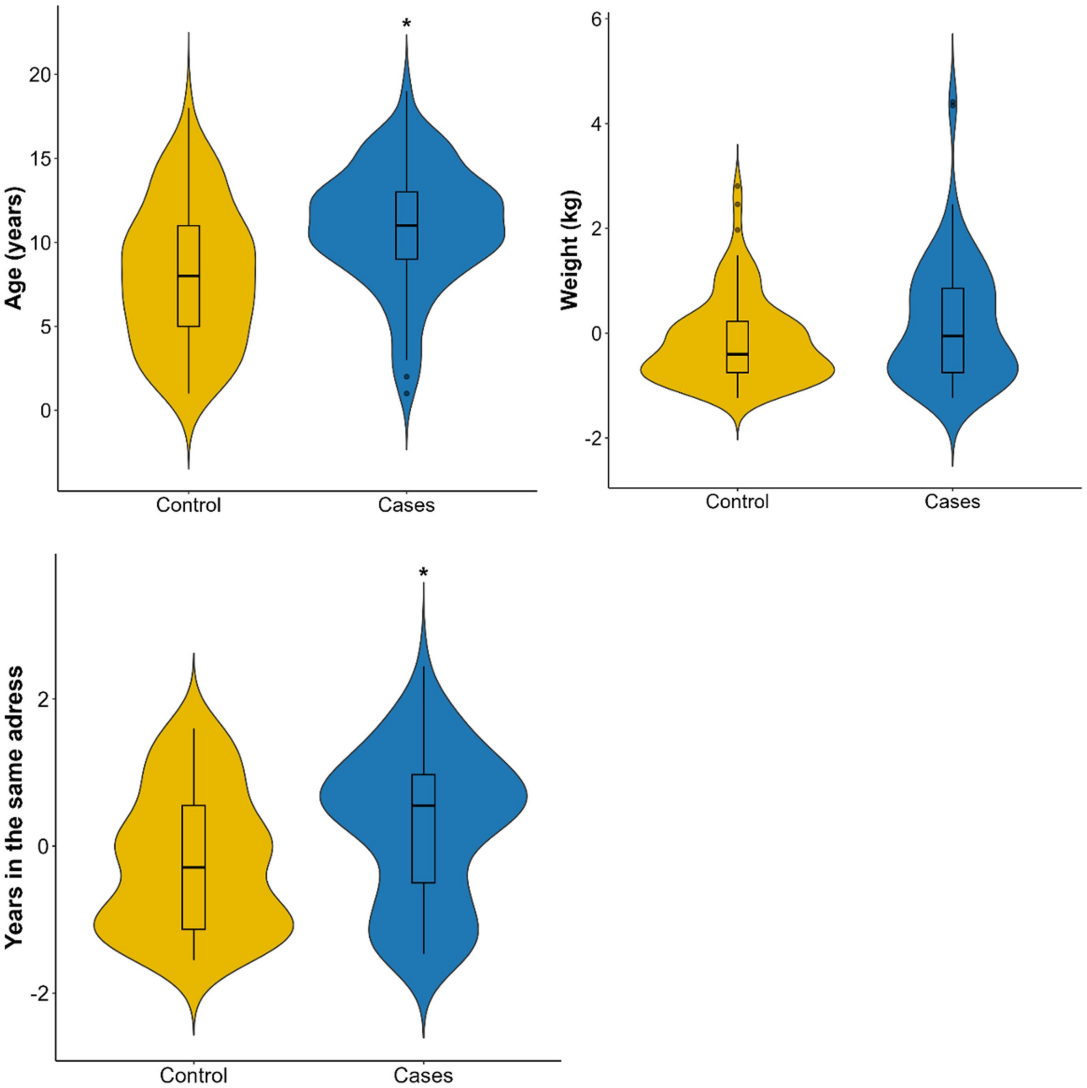
Distribution of age (years), weight (kg), and duration of residence at the same address (years) in cases (*n* = 80) and controls (*n* = 95).

Figure 2 shows the distribution of age and weight by group. Dogs with tumors had a median age of 11 years, which was significantly older than the median of 7 years observed in the control group (*p* < 0.001, Wilcoxon test). However, weight did not differ significantly between the two groups (*p* = 0.089). Among the affected dogs, 43% were small breeds (0–15 kg) and 34% were large breeds (25–45 kg).

Table 2 summarizes the descriptive analysis and univariate logistic regression for the categorical variables. The distribution of males and females was balanced, with no significant association between sex and tumor development (*p* = 0.634). However, reproductive status was significantly associated with tumor risk, as intact dogs showed higher odds of disease. Environmental exposures were also significant: exposure to barbecue smoke increased the risk, while exposure to incense and the consumption of mineral water were associated with a reduced risk. Regarding dietary habits, dogs on a mixed diet were more frequently affected, whereas those consuming fruits and vegetables had a significantly lower risk. Notably, a history of periodontal disease and prior professional dental cleaning were associated with increased tumor risk.

**Table 2:** Descriptive analysis and univariate logistic regression results of categorical variables.

Variables	Control (*n*, %)	Cases (*n*, %)	Univariate logistic regression		
			Univariate OR	95% CI	*p*-value
Sex	95	80			
Female	58 (61.1%)	46 (57.5%)	Ref		
Male	37 (38.9%)	34 (42.5%)	1.16	0.63–2.13	0.634
Reproductive status					
Intact	14 (14.7%)	23 (28.7%)	Ref		
Neutered	81 (85.3%)	57 (71.2%)	0.43	0.20–090	**0.024**
Breed					
Mixed-breed	43 (45.3%)	32 (40%)	Ref		
Purebreed	52 (54.7%)	48 (60%)	1.24	0.68–2.27	0.483
Top 7 breeds (*n* > 5)					
Labrador Retriever	3 (3.1%)	8 (10.0%)	3.58	0.95–17.35	0.074
Poodle	5 (5.2%)	4 (5.0%)	1.07	0.24–4.37	0.918
Golden Retriever	5 (5.2%)	3 (3.7%)	0.80	0.15–3.53	0.778
Lhasa Apso	5 (5.2%)	2 (2.5%)	0.53	0.07–2.67	0.474
Dachshund	3 (3.1%)	3 (3.7%)	1.34	0.23–7.67	0.727
Shitzu	4 (4.2%)	2 (2.5%)	0.67	0.08–3.66	0.657
Yorkshire Terrier	4 (4.2%)	2 (2.5%)	0.67	0.08–3.66	0.657
Zone					
Rural	1 (1.1%)	8 (10.0%)	Ref		
Urban	94 (98.9%)	72 (90.0%)	0.1	0.01–0.54	**0.029**
Feeding					
Commercial	69 (72.6%)	36 (45.0%)	0.37	0.10–1.25	0.112
Mixed	21 (22.1%)	37 (46.3%)	1.26	0.34–4.45	0.722
Homemade	5 (5.3%)	7 (8.7%)			
Fruits and vegetables	50 (52.6%)	23 (28.7%)	0.36	0.19–0.68	**0.002**
Exposed to					
Incense	33 (34.7%)	11 (13.7%)	0.3	0.13–0.63	**0.002**
Tobacco smoke	18 (18.9%)	11 (13.7%)	0.68	0.29–1.53	0.359
Barbecue smoke	10 (10.5%)	18 (22.5%)	2.47	1.08–5.91	**0.035**
Drinking					
Mineral water	36 (37.9%)	19 (23.7%)	0.51	0.26–0.98	**0.046**
Tap water	73 (76.8%)	67 (83.7%)	1.55	0.73–3.40	0.257
Dental hygiene					
Toothbrushing	7 (7.4%)	6 (7.5%)	1.02	0.32–3.20	0.974
Dental cleaning	33 (34.7%)	42 (52.5%)	2.08	1.13–3.84	**0.019**
Periodontal disease history	28 (29.5%)	40 (50.0%)	2.39	1.29–4.49	**0.006**

OR, odds ratio; CI, confidence interval; Ref, reference group. Statistical significance was set at *p* < .05. Values in bold indicate statistical significance (*p* < 0.05) based on the univariate logistic regression analysis.

### 3.3 Multivariate logistic regression analysis

The multivariate logistic regression model included variables significant in the univariate analysis to adjust for potential confounders. Table 3 presents the final model results. Older dogs and cães with higher body weight had significantly higher odds of developing oral tumors. Exposure to barbecue smoke remained a significant risk factor (aOR = 2.98; 95% CI: 1.13–8.41; *p* = 0.032), whereas exposure to incense showed a statistically significant inverse association (aOR = 0.34; 95% CI: 0.13–0.82; *p* = 0.019).

**Table 3:** Multivariate logistic regression analysis of risk factors for solid oral tumors in dogs.

Variables	Adjusted Odds Ratio (aOR)	95% CI	*p*-value
Variables			
Age	1.86	1.20–2.98	0.007
Weight	1.64	1.11–2.52	0.016
Mineral water	0.45	0.18–1.08	0.079
Urban zone	0.2	0.01–1.43	0.166
Exposition to barbecue	2.98	1.13–8.41	0.032
Exposition to incense	0.34	0.13–0.82	0.019
Commercial food	0.63	0.13–2.70	0.532
Mixed food	2.22	0.46–10.29	0.308
Fruits and vegetables	0.55	0.24–1.24	0.152
Dental cleaning	2.43	1.00–6.15	0.055

aOR, adjusted odds ratio; CI, confidence interval. The model was optimized using the Akaike Information Criterion (AIC).

Professional dental cleaning was marginally associated with higher risk (aOR = 2.43; *p* = 0.055). This association should be interpreted with caution; it likely reflects a surveillance bias, where dogs receiving regular dental interventions undergo more frequent oral examinations, leading to a higher rate of tumor detection rather than the procedure itself promoting oncogenesis.

## 4 Discussion

This study examined the epidemiological and etiological factors associated with the development of solid oral tumors in dogs. Age emerged as a major risk factor, with affected dogs being significantly older than healthy counterparts. This aligns with previous research showing that cancer is largely a disease of aging, influenced by the accumulation of genetic mutations, diminished DNA repair capacity, and persistent inflammatory processes over time (14–16).

Body size was also associated with a higher risk of oral tumors. This finding supports earlier evidence that larger breeds have greater cancer susceptibility, potentially due to accelerated cell turnover and prolonged exposure to growth factor signaling, both of which contribute to oncogenic transformation (17). Reproductive status was significantly associated with oral tumor development, with intact dogs demonstrating a higher risk than neutered dogs. A plausible explanation is that intact dogs may experience different lifestyle patterns, including reduced veterinary care and fewer preventive interventions, which could contribute to later-stage diagnoses. Furthermore, hormonal influences on oral mucosal tissue may differ from those documented in mammary or prostate tumors, underscoring the need for further investigation into the role of sex hormones in oral oncogenesis.

Environmental exposures were also significant predictors, with barbecue smoke showing a positive association with oral tumor development. This finding is consistent with the well-documented carcinogenic potential of polycyclic aromatic hydrocarbons (PAHs) released during the combustion of organic material (18). PAHs have been strongly associated with multiple cancers in humans and animals, and their presence in airborne barbecue smoke may contribute to oral mucosal irritation and chronic inflammation, creating a pro-tumorigenic environment. Prolonged exposure to these compounds may also promote the accumulation of DNA damage in oral epithelial cells.

An unexpected and paradoxical finding was the apparent protective effect of incense exposure against oral tumors. This result appears counterintuitive, as incense burning releases volatile organic compounds and particulate matter, several of which are recognized carcinogens (12). However, this association should be framed cautiously. Incense exposure may function as a proxy for other unmeasured lifestyle variables—such as specific hygiene practices, household stress levels, or dietary patterns—that influence cancer susceptibility. This likely represents residual confounding rather than a biological protective effect. Further studies with more detailed exposure assessments are required to clarify this relationship.

Similarly, the consumption of mineral water was associated with a reduced risk. This may indicate a protective role of specific minerals or the absence of harmful contaminants found in tap water, such as heavy metals and chlorine byproducts (13). Nevertheless, this finding may also reflect confounding by socioeconomic factors, as owners providing bottled water may engage in other health-conscious practices, such as offering higher-quality diets and ensuring more consistent veterinary care.

Dietary habits were significant determinants; dogs fed a mixed diet demonstrated a higher likelihood of tumors, whereas those consuming fruits and vegetables exhibited a lower risk. The risk from mixed diets may stem from processed human foods containing preservatives or additives with carcinogenic potential (19, 20). Conversely, the protective effect of fruits and vegetables supports evidence that bioactive compounds like polyphenols mitigate oxidative stress (21, 22).

Finally, the association between periodontal disease and professional dental cleaning and increased tumor risk may represent reverse causality or surveillance bias. Dogs receiving frequent dental procedures often have chronic inflammation, a well-established precursor to neoplasia (23, 24). Therefore, this association likely reflects enhanced veterinary surveillance in dogs predisposed to oral pathology rather than a direct tumor-promoting effect of dental procedures.

Overall, these findings emphasize the multifactorial nature of canine oral tumors. Due to the observational design, causality cannot be confirmed, and limitations such as recall bias and residual confounding must be acknowledged. Future research should prioritize prospective cohort studies to validate these associations.

## 5 Conclusion

These findings underscore the importance of preventive strategies in veterinary oncology. Minimizing exposure to established carcinogens, optimizing dietary practices, and promoting oral health may be critical in reducing cancer risk. Additionally, the unexpected protective associations observed for certain exposures highlight the need for a more nuanced and cautious understanding of environmental contributions to cancer biology. Because of the observational nature of this study, these results should be viewed as significant associations rather than confirmed causal links. Integrating epidemiological evidence with molecular research will be essential for future studies to advance the understanding of canine oral tumor etiology and to guide the development of more effective prevention and treatment strategies.

## Data Availability

The raw data supporting the conclusions of this article will be made available by the authors, without undue reservation.

## Footnotes

1 https://forms.gle/7o8siWDDt4SafG7w9.
